# Preparation and Short-Term Aging Properties of Asphalt Modified by Novel Sustained-Release Microcapsules Containing Rejuvenator

**DOI:** 10.3390/ma12071122

**Published:** 2019-04-04

**Authors:** Xin Yan, Guotao Ning, Xiaofeng Wang, Tao Ai, Peng Zhao, Zhenjun Wang

**Affiliations:** 1 School of Materials Science and Engineering, Chang’an University, Xi’an 710064, China; xinyan@chd.edu.cn (X.Y.); aitao@chd.edu.cn (T.A.); zyzhaop@chd.edu.cn (P.Z.); 2 Engineering Research Center of Pavement Materials, Ministry of Education of P.R. China, Chang’an University, Xi’an 710061, China; 3 Research and Development Center of Transport Industry of Technologies, Materials and Equipments of Highway Construction and Maintenance, Zhengzhou 450052, China; wangxf0351@sina.com

**Keywords:** modified asphalt, sustained-release microcapsules, rejuvenator, aging properties

## Abstract

Aged asphalt can enormously affect the performance of asphalt pavement and cause serious environmental hazards. Microcapsule-modified asphalt is one of the effective means to improve the anti-aging ability of asphalt. In this work, novel sustained-release microcapsules containing rejuvenator were prepared by the solvent evaporation method. The morphology of the microcapsules was characterized by scanning electron microscopy (SEM). The sustained-release properties of the microcapsules were investigated by static thermogravimetric analysis. The physical properties such as penetration, ductility, softening point, and Brookfield viscosity of the original asphalt and microcapsule-modified asphalt were studied. In addition, the viscoelasticity of the original asphalt and microcapsule-modified asphalt was investigated by means of a dynamic shear rheometer (DSR). The results show that the prepared microcapsules have a smooth surface and a complete encapsulation with an average particle size of 60 μm. After the heating treatment (above 140 °C), a large number of micropores were formed on the shell surface of microcapsules, which provided a structural basis for the sustained-release of rejuvenator. The release rate of the rejuvenator was obviously slowed down by the microcapsules. The aging behavior of sustained-release microcapsules containing rejuvenator-modified asphalt can be greatly improved. The enhanced anti-aging properties of sustained-release microcapsule-modified asphalt are attributed to the functions of the rejuvenator which can be slowly released from the micropores on the microcapsules’ surface, after which the light components lost in the original asphalt can be supplemented.

## 1. Introduction

Asphalt pavement has become the main type of road pavement because of its advantages of comfortable driving, smooth surface, wear-resistance, and convenient maintenance, which is widely used in urban road construction and airport construction [1,2]. However, in the long-term service of asphalt pavement, the aging of asphalt pavement is inevitable due to the change of the external environment. The aged asphalt pavement would become brittle, and micro-cracks would generate under cyclic load, which eventually lead to the destruction of the asphalt pavement.

In order to delay the aging process and extend the service life of asphalt pavement, a great deal of research has been done on the performance recovery of aged asphalt. More studies have proved that the volatilization of light components (saturates and aromatics) in asphalt is one of the main causes of asphalt aging [3,4,5,6]. If the chemical composition of asphalt can be improved in time during the aging process, the aging speed of asphalt can be effectively delayed. Rejuvenator with a high content of maltenes is widely used in asphalt regeneration by regulating the chemical composition of asphalt. There are two widely used regeneration technologies, namely fog seal technology and asphalt pavement recycling technology, that can add rejuvenator into aged asphalt mixtures [7,8]. However, there are still some shortcomings in these two technologies, which are mainly manifested in the need to interrupt traffic and human intervention in the repair process. Encapsulation technology provides a promising approach to solve these problems [9,10,11]. The microcapsules containing rejuvenator were added to the asphalt. The propagation of the micro-cracks leads to the rupture of the microcapsules, which release the rejuvenator into the micro-cracks. The released rejuvenator not only restores the asphalt stiffness and strength by closing the micro-cracks but also promotes the self-healing properties of asphalt, thus extending the serviceability time of asphalt pavements. The key to microcapsule crack repair is that the micro-cracks must be able to extend to the microcapsules; otherwise, the microcapsules cannot be ruptured to release the rejuvenator [12,13,14,15,16]. By investigating the characteristics of microcapsules, we found that there are two kinds of microcapsule release mechanisms—one is the rapid release after the capsule wall ruptures, and the second is the slow release through the pores of the capsule wall [17,18]. If the microcapsule with slow-release rejuvenator is added to the asphalt, it can delay the aging of asphalt without triggering the micro-cracks to cause microcapsule rupture, thereby greatly prolonging the service life of asphalt pavements.

In this work, novel sustained-release microcapsules containing rejuvenator were prepared by the solvent evaporation method. The morphology of the microcapsules was characterized by scanning electron microscopy. The sustained-release properties of the microcapsules were investigated by thermogravimetric analysis. The penetration, ductility, softening point, and Brookfield viscosity of microcapsule-modified asphalt and original asphalt samples were measured. The complex shear modulus (G*) and phase angle (δ) of microcapsule-modified asphalt and original asphalt were investigated by means of a dynamic shear rheometer (DSR).

## 2. Materials and Methods

### 2.1. Raw Materials

The core material used as the rejuvenator was a dense, aromatic oil (density is 1.050 g/cm^3^ and viscosity is 10.0 Pa·s at 20 °C). The shell material was commercial polymethyl methacrylate (PMMA). Gelatin (GE), sodium dodecylbenzenesulfonate (SDBS), octyl-phenyl polyoxyethylene ether (OP-10), sodium dodecyl sulfate (SDS), methylene chloride, and other chemical reagents were of analytical grade. The properties of the original asphalt used in this work are shown in Table 1.

### 2.2. Preparation of Sustained-Release Microcapsules

**Figure 1:**
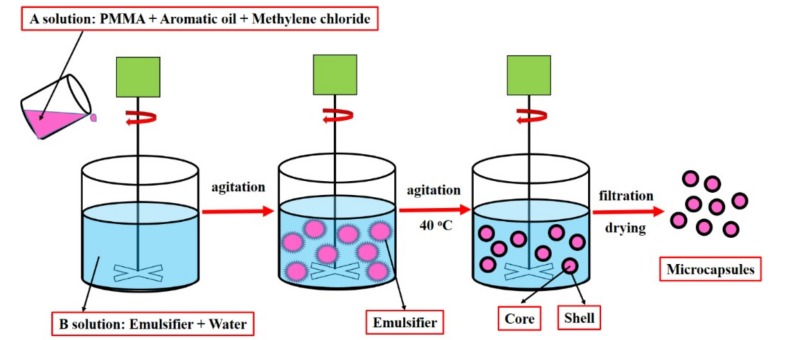
The schematic illustration of the sustained-release microcapsules preparation.

In this work, sustained-release microcapsules were prepared by the solvent evaporation method [22]. The core material used as the rejuvenator was aromatic oil, and the shell material was PMMA. The emulsifiers used were GE, SDBS, SDS, and OP-10, respectively. The preparation process is described as follows—solution A: PMMA and aromatic oil were added to 20 mL methylene chloride and mixed for 2 h. Solution B: emulsifier was added to 200 mL of water and mixed for 1 h. Then solution A was added dropwise to solution B under a stirring speed of 700 r/min for 30 min. Then the mixed solution was heated to 40 °C with stirring until the complete volatilization of methylene chloride was achieved. At last, the resultant microcapsules were filtered, washed with pure water, and dried at 80 °C for 4 h. The schematic illustration of the sustained-release microcapsules preparation is shown in Figure 1.

### 2.3. Preparation and Aging Test of Microcapsule-Modified Asphalt

**Figure 2:**
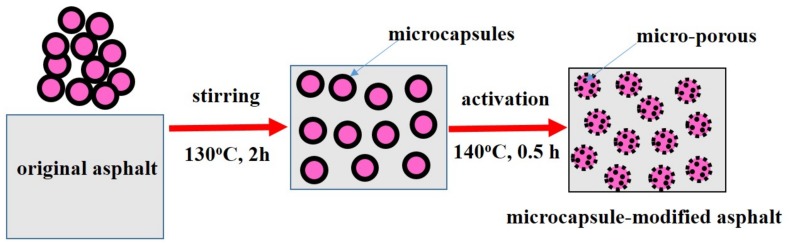
The schematic illustration of the microcapsule-modified asphalt preparation.

The preparation process of microcapsule-modified asphalt is as follows—microcapsules with a mass ratio of 5% (by the weight of asphalt) were added to the asphalt, and the mixture was heated to 130 °C and stirred for 2 h to make the microcapsules evenly distributed in the asphalt. The mixture was then heated to 140 °C for 0.5 h so that the microcapsules were activated to obtain microcapsule-modified asphalt. The schematic illustration of the preparation of the microcapsule-modified asphalt is shown in Figure 2. The original asphalt and microcapsule-modified asphalt were short-term aged by the Rolling Thin Film Oven Test (RTFOT). The detailed RTFOT procedure is as follows—the asphalt samples were placed in cylindrical glass bottles and then the bottles were transferred to a rotating carriage in the oven. The carriage rotated in the oven at a temperature of 163 °C, and the bottles were held for different aging times (1, 2, 3, and 4 h). Finally, original asphalt, microcapsule-modified asphalt, different aging time original asphalt, and different aging time microcapsule-modified asphalt samples were stored for physical properties tests.

### 2.4. Characterization of Microcapsules

The surface morphology of microcapsules was characterized by scanning electron microscopy (SEM, Hitachi S-4800, Tokyo, Japan). In order to enhance the conductivity of the microcapsule samples, they were sprayed with gold before the test. The accelerating voltage of SEM was 3.0 kV.

The coating rate was used to characterize the efficiency of the synthesis method, which can be calculated by Equation (1):

(1) $$\mathrm{CR}=\frac{M_{2}}{M_{1}}\times 100\%$$

where CR is the coating rate of microcapsules, *M_2_* is the mass of the aromatic oil which was successfully capsuled in the microcapsule, and *M_1_* is the total mass of the aromatic oil added in the test.

### 2.5. Characterization Methods of Asphalt

In this work, physical property tests including penetration at 25 °C, softening point, ductility at 15 °C, and viscosity at 135 °C were conducted on original asphalt and modified asphalt before and after RTFOT according to ASTM D5 [19], ASTM D36 [21], ASTM D113 [20], and ASTM D4402 [23], respectively. The dynamic shearing rheological tests of original asphalt and microcapsule-modified asphalt before and after aging were carried out by a Bohlin Gemini 2 ADS Dynamic Shear Rheometer (DSR, Cirencester, UK).

The aging degree of asphalt can be characterized by the aging index which refers to the ratio or the difference of physical or chemical indexes of asphalt before and after aging. The penetration ratio (PR), softening point increment (ΔT), ductility ratio (DR), and viscosity aging index (VAI) are commonly used evaluation indexes and can be calculated by Equations (2)–(5), respectively:

(2) $$\mathrm{PR}=\frac{P_{2}}{P_{1}}\times 100\%$$

where *P*_1_ and *P*_2_ are the samples’ penetrations before and after RTFOT, respectively,

(3) $$∆T=T_{2}-T_{1}$$

where *T*_1_ and *T*_2_ are the samples softening points before and after RTFOT, respectively,

(4) $$\mathrm{DR}=\frac{D_{2}}{D_{1}}\times 100\%$$

where *D*_1_ and *D*_2_ are the samples ductility before and after RTFOT, respectively,

(5) $$\mathrm{VAI}=\mathrm{lg}\left[\mathrm{lg}\left({ƞ}_{2}\times 1000\right)\right]-\mathrm{lg}\left[\mathrm{lg}\left({ƞ}_{1}\times 1000\right)\right],$$

where η_1_ and η_2_ are the viscosity before and after RTFOT, respectively.

## 3. Results and Discussion

### 3.1. Coating Ratio of Microcapsules

#### 3.1.1. Effects of Different Emulsifiers

The emulsification of the oil phase in the water phase has an important effect on the coating ratio of microcapsules by the solvent evaporation method [24,25]. Therefore, the selection of suitable aqueous emulsifiers is crucial to the successful encapsulation. In this study, different emulsifiers, such as sodium dodecylbenzene sulfonate (SDBS), sodium dodecyl sulfate (SDS), gelatin (GE), and OP-10, were selected as core materials to emulsify the rejuvenator. The mass ratio of the core material to shell material was kept at 1:1.3 in the experiment.

**Figure 3:**
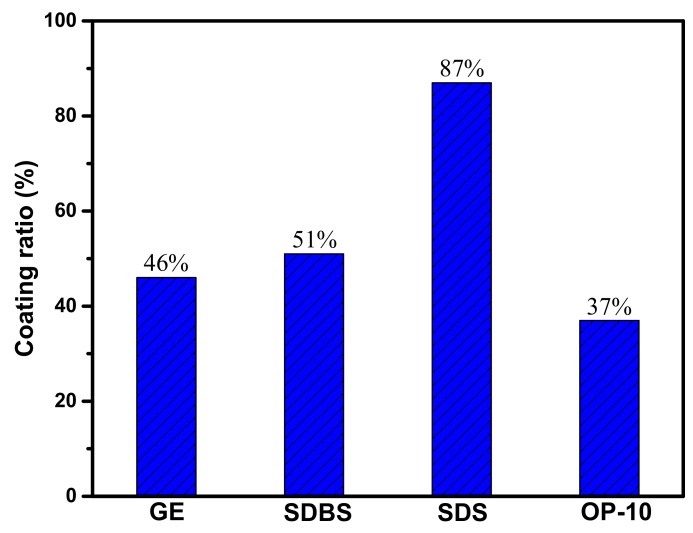
Coating ratio of microcapsules with different emulsifiers.

Figure 3 shows the comparison of the coating rate of microcapsules with different emulsifiers. The microcapsules with a high coating rate could be obtained when SDS is used as the emulsifier. The other three emulsifiers did not produce an effective coating on aromatic oils. The results indicate that the high surface activity of SDS can contribute to the uniform dispersion of liquid aromatic oil droplets into the continuous phase.

#### 3.1.2. Effects of Core/Shell Mass Ratios

**Figure 4:**
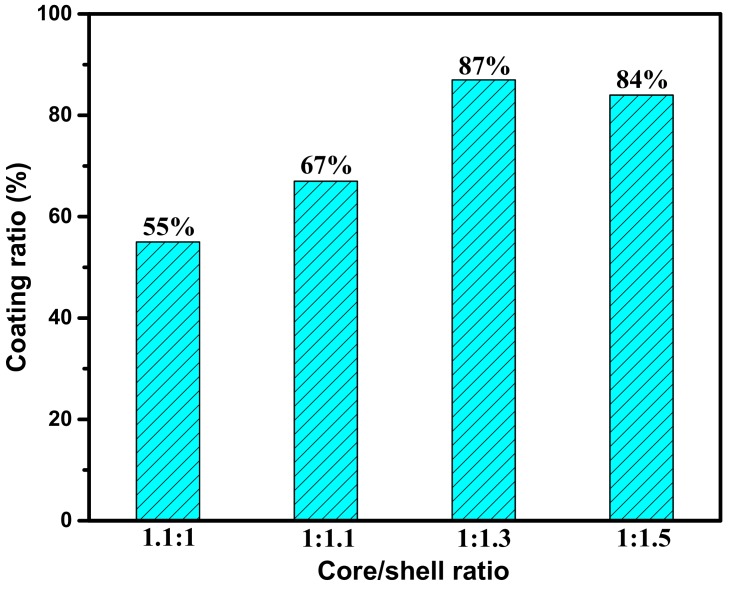
Coating ratio of microcapsules with different core/shell mass ratios.

In the process of microencapsulation, the mass ratio of the core material to the shell material is an important factor affecting the coating ratio of microcapsules [26,27]. Figure 4 shows the comparison of the coating ratios of microcapsules with different core/shell mass ratios. The SDS was used as the emulsifier in the experiment. When the core/shell mass ratio was 1.1:1, the coating ratio of the microcapsules was low because of insufficient shell material. When the core/shell ratio was increased to 1:1.1, the coating ratio of the microcapsules was improved. The result shows that the coating ratio of microcapsules was the highest when increasing the core/shell to 1:1.3 and no significant increase was achieved by adding more shell material (1:1.5).

### 3.2. Sustained-Release Performance of Microcapsules

#### 3.2.1. Morphology Analyses

**Figure 5:**
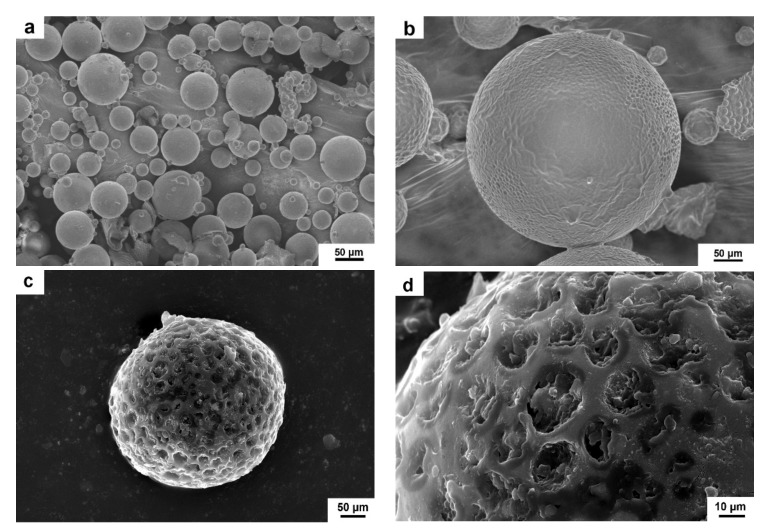
SEM images of sustained-release microcapsules (**a**); microcapsules after heating at 130 °C (**b**); and microcapsules after heating at 140 °C (**c**,**d**).

Figure 5 shows the SEM surface morphologies of microcapsules. It can be seen that the average particle size of microcapsules is about 60 μm. A smooth surface and complete encapsulation are seen in Figure 5a. In order to observe the shell change of microcapsules, the microcapsules were heated at 130 °C and 140 °C for 1 h, separately. In Figure 5b, the shell surface morphology of the microcapsules did not change significantly and remained completely encapsulated. The results show that microcapsules possess excellent thermal stability in the range below 130 °C. With a further increase of the temperature to 140 °C, the shell surface morphology of the microcapsules became coarser compared to Figure 5c. A large number of micropore structures can be clearly seen in the shell of the microcapsules in the enlarged SEM image (Figure 5d). The results show that the micropore structure provides favorable conditions for the slow release of rejuvenator.

#### 3.2.2. Sustained-Release Performance

**Figure 6:**
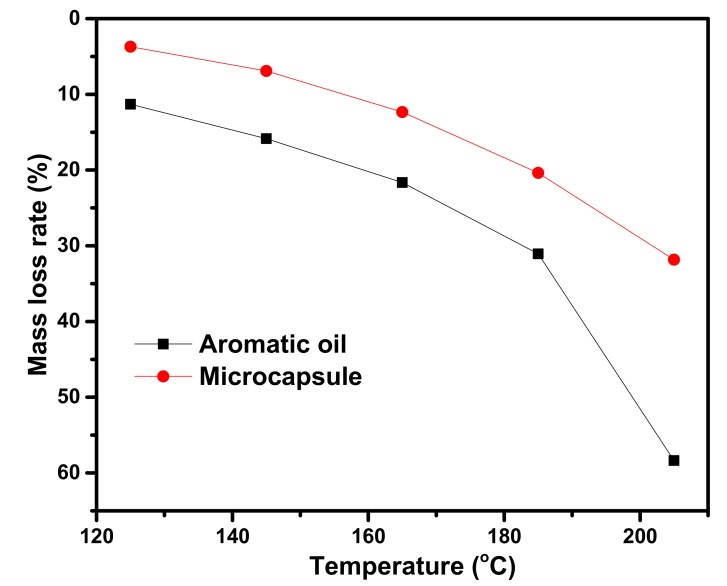
The mass loss rate of aromatic oil and aromatic oil microcapsules at different temperatures.

In order to investigate the sustained-release behavior of microcapsules, the mass loss experiment was investigated by static thermogravimetric analysis. Figure 6 shows the mass loss of aromatic and aromatic oil microcapsules at different temperatures for 4 h. When the heating temperatures were 125, 145, 165, 185, and 205 °C, the total mass loss rates of aromatic oil were 11%, 16%, 21%, 31%, and 58%, respectively. However, the mass loss rates of aromatic oil microcapsules at the same temperatures were 4%, 7%, 12%, 20%, and 32%, respectively.

It can be seen that the aromatic oil and aromatic oil microcapsules are volatile at different temperatures, but the mass loss rate of aromatic oil is significantly higher than that of the aromatic oil microcapsules at the same temperature. The results show that aromatic oil microencapsulation can significantly reduce the volatility of aromatic oils, which enables the aromatic oil to play its role for a long time.

### 3.3. Physical Properties of Different Asphalt after Aging

#### 3.3.1. Penetration, Softening Point, and Ductility of Asphalt

The original asphalt and obtained microcapsule-modified asphalt were subjected to RTFOT aging at different times, and the residue was collected and tested for penetration, softening point, ductility, and Brookfield viscosity, respectively.

**Figure 7:**
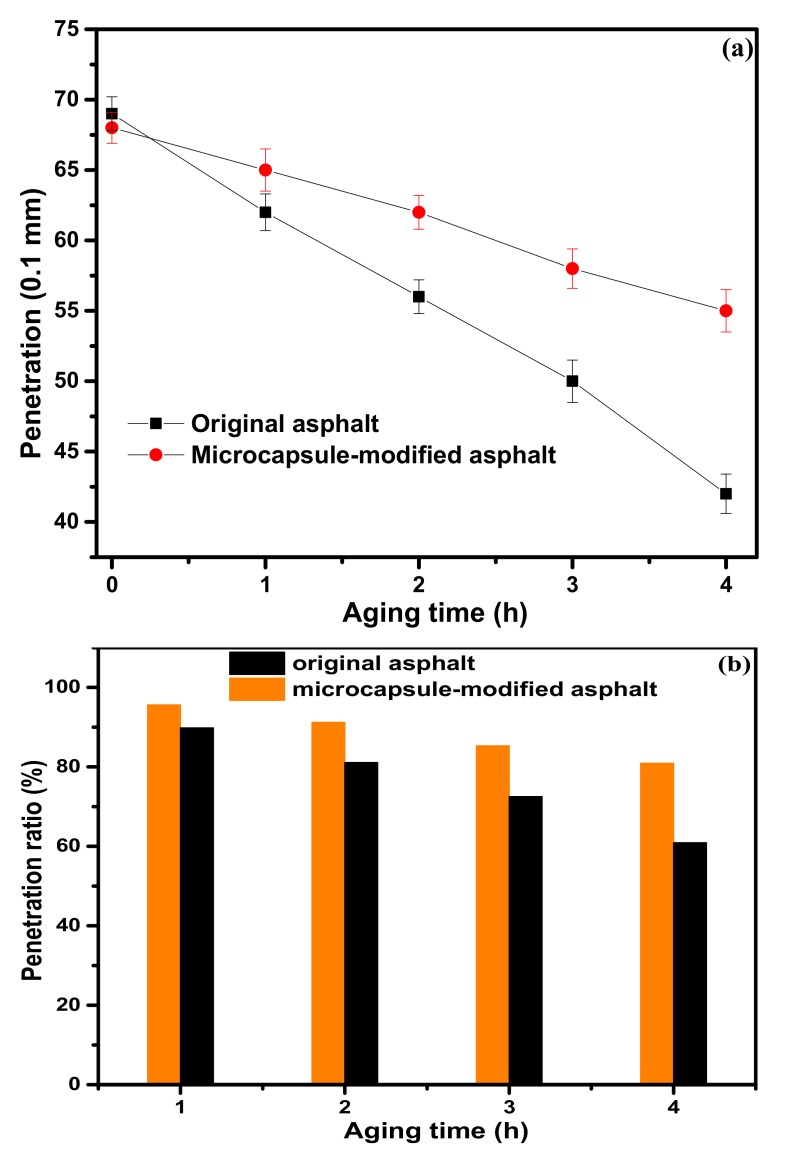
The penetration versus the aging time of different asphalt samples (**a**); the penetration ratio versus the aging time of different asphalt samples (**b**).

The penetration versus the aging time of the original asphalt and microcapsule-modified asphalt is shown in Figure 7a. From Figure 7a, the penetration of the original asphalt and the microcapsule-modified asphalt samples decreased with increased aging time. The result was analyzed according to the change of asphalt composition. The asphalt can be divided into saturates, aromatics, resins, and asphaltenes by four composition analysis methods. During the aging process, saturates and aromatics composition were easily volatilized from the asphalt [28,29]. Hence, the asphalt becomes stiffer due to the increase of the resins and asphaltenes content. Compared with the original asphalt, the penetration of microcapsule-modified asphalt decreased slowly, as shown in Figure 7a. The results show that the slow release of the rejuvenator from the microcapsule makes up for the volatilization of the light component in the asphalt, thus slowing down the aging of the asphalt. The penetration ratio was calculated to analyze the aging resistance performance of the asphalt using Equation (2) and the results are shown in Figure 7b. It can be concluded that the penetration ratio of the microcapsule-modified asphalt after aging is higher than that of the original asphalt. The results further confirmed that the aging of the asphalt was slowed down by the addition of microcapsules.

**Figure 8:**
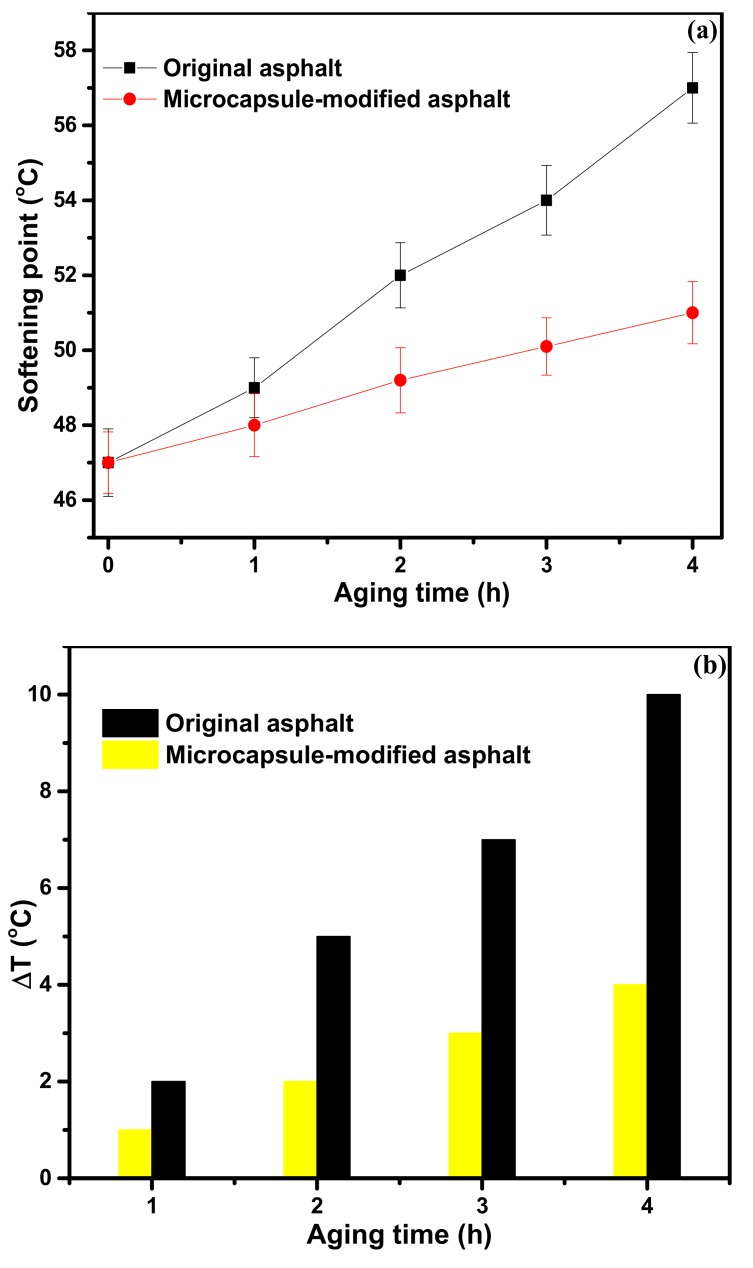
The softening point versus the aging time of different asphalt samples (**a**); the increment of softening point (Δ*T*) versus the aging time of different asphalt samples (**b**).

The softening point of the original asphalt and microcapsule-modified asphalt with different aging times is shown in Figure 8a. The results show that the softening point of the two kinds of asphalt increases after short-term aging. Many studies show that the increase in the softening point of asphalt is mainly caused by the transformation of asphalt components [30]. Nevertheless, the speed softening point increase of the original asphalt is much faster than that of the microencapsulated asphalt. The softening point increment was calculated to analyze the aging resistance performance of asphalt using Equation (3) and the results are shown in Figure 8b. The softening point increment of the original asphalt after aging is higher than that of the microcapsule-modified asphalt, indicating that the aging degree of the original asphalt is higher than that of the microcapsule-modified asphalt.

**Figure 9:**
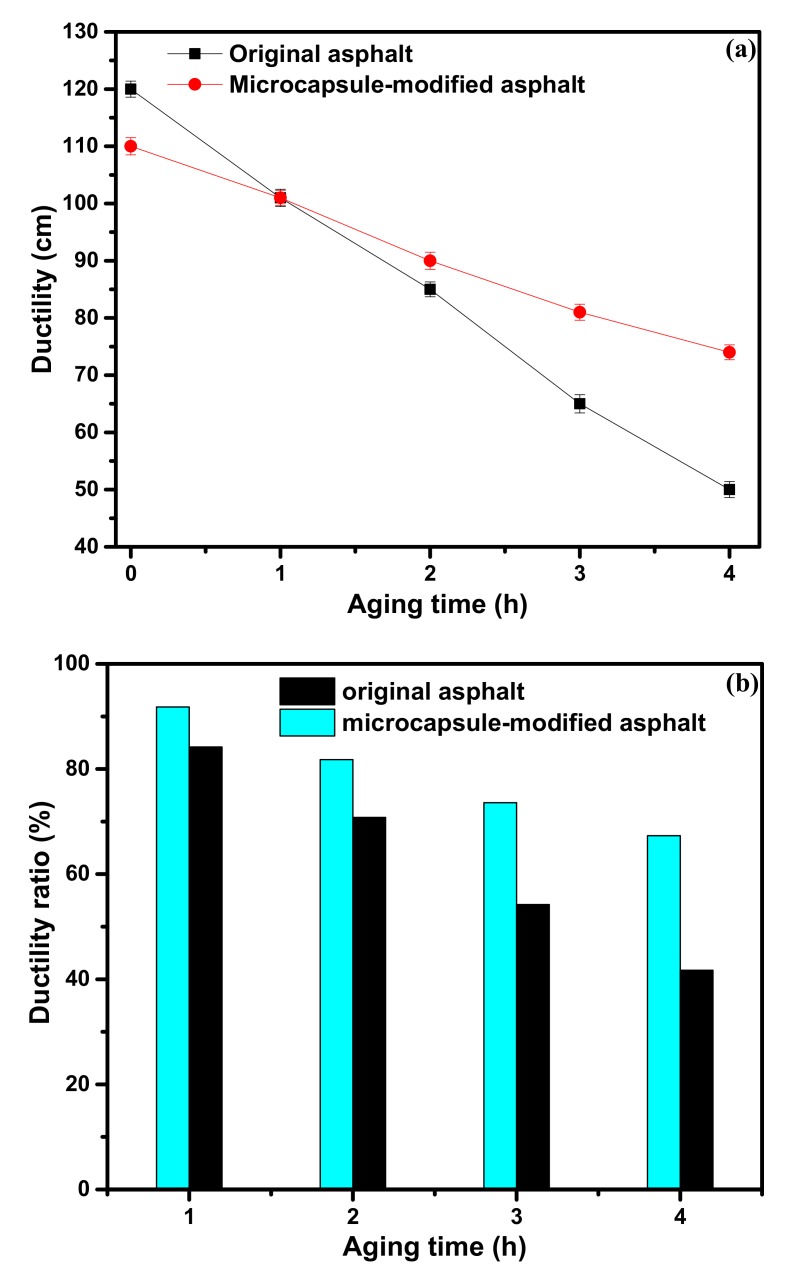
The ductility versus the aging time of different asphalt samples (**a**); the ductility ratio versus aging time of different asphalt samples (**b**).

The ductility of asphalt is an important index to evaluate the flexibility of asphalt [31]. The ductility versus aging time of the original asphalt and microcapsule-modified asphalt is shown in Figure 9a. The ductility of the original asphalt after aging for 4 h was greatly reduced to 50.2 cm. The ductility of the microcapsules-modified asphalt after aging for 4 h was reduced to 70.2 cm, which is higher than that of the original asphalt after aging. The ductility ratio of asphalt was calculated to analyze the aging resistance performance of asphalt using Equation (4), and the results are shown in Figure 9b. The ductility ratio of the microcapsule-modified asphalt is far higher than that of the original asphalt, which shows better plasticity of the modified asphalt after the short-term aging. The results indicate that a certain amount of aromatic oil is released during short-term aging to make up for the volatile light components of asphalt so that the asphalt short-term thermal oxygen aging resistance is improved.

#### 3.3.2. Brookfield Viscosity of Asphalt

**Figure 10:**
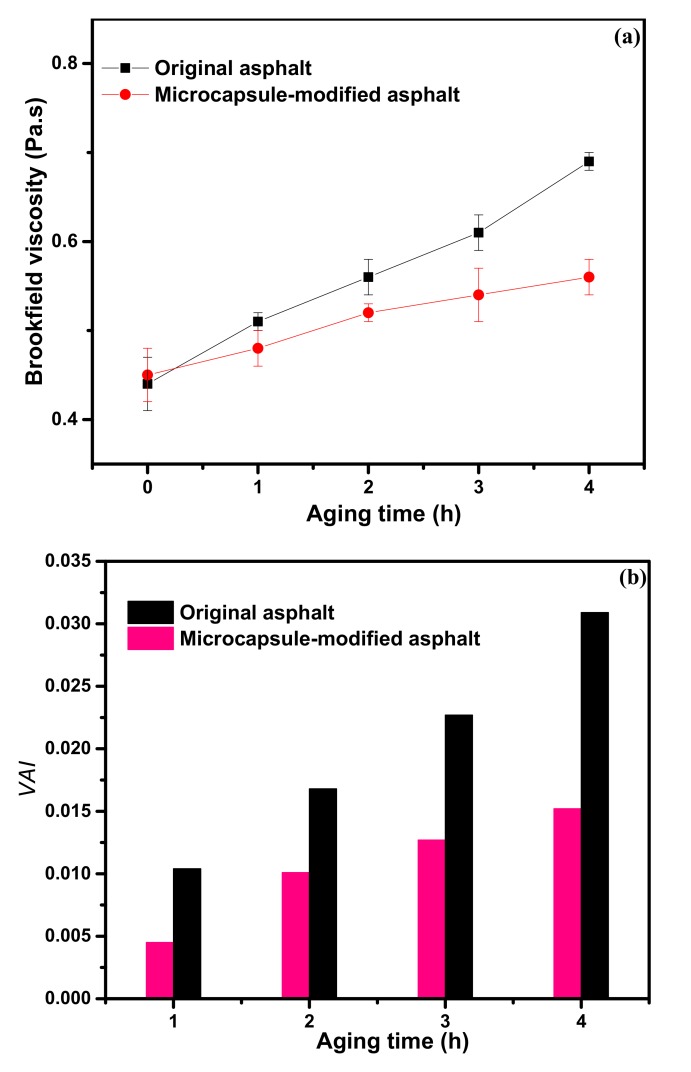
Brookfield viscosity versus the aging time of different asphalt samples (**a**); the viscosity aging index (VAI) versus aging time of different asphalt samples (**b**).

Viscosity is an important index affecting the application of asphalt. If the viscosity is too high, it is difficult to blend and compact. Hence, it is very important to control asphalt viscosity in a reasonable time period [32,33,34]. In order to study the effect of aging on the viscosity of asphalt, rotational viscosities of the original asphalt and microcapsule-modified asphalt were tested at 135 °C. Figure 10a shows the results of the viscosity tests. The viscosity of the original asphalt and microcapsule-modified asphalt increased with increased aging time. However, the rate at which the viscosity of the original asphalt increased was much faster than that of the microcapsule-modified asphalt. This shows that the microcapsule-modified asphalt can reduce the effect of thermal oxidation aging, which inhibits the increase of the viscosity of asphalt effectively. The viscosity aging index (VAI) is a common index, which is used to evaluate the aging degree of asphalt after RTFOT. From the VAI index calculated using Equation (5), the VAI of original asphalt was far higher than that of the microcapsule-modified asphalt, which shows that the microcapsules have an evident effect on slowing down the short-term aging degree of asphalt.

#### 3.3.3. Viscoelasticity of Microcapsule-Modified Asphalt

**Figure 11:**
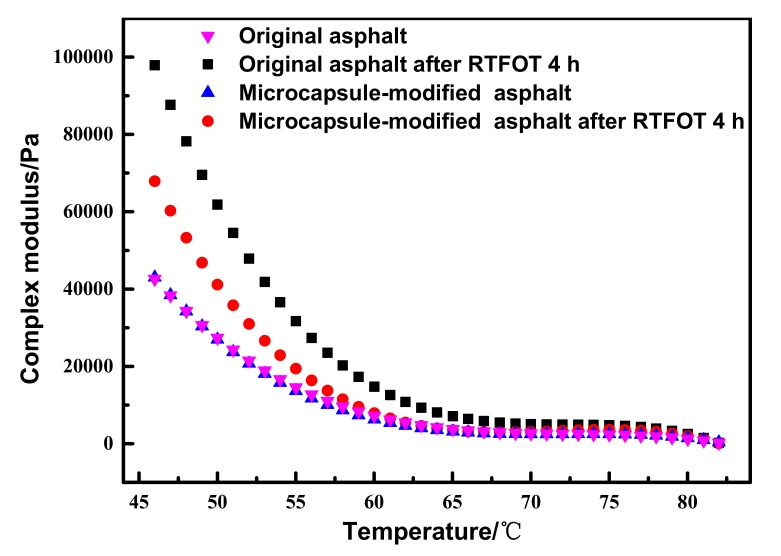
The complex modulus of different asphalt samples before and after Rolling Thin Film Oven Test (RTFOT) aging.

The change of the complex shear modulus G* and phase angle δ due to the aging of asphalt were characterized by dynamic shear rheometry (DSR). At the same time, the change of asphalt viscoelasticity was analyzed. Before and after short-term aging of asphalt, samples were tested in a temperature range of 46–82 °C. The results of complex shear modulus G* before and after RTFOT aging of different asphalt samples are shown in Figure 11.

As can be seen from Figure 11, all asphalt complex modulus G* values decreased with increasing temperature, which is due to the asphalt transition from low to high temperature; the rheological state then changed from the elastic state to the viscous flow state. In contrast to the original asphalt, the increase of complex modulus G* caused by aging of the microcapsule-modified asphalt was greatly reduced. The results indicate that addition of microcapsules can effectively improve the anti-aging properties of asphalt.

**Figure 12:**
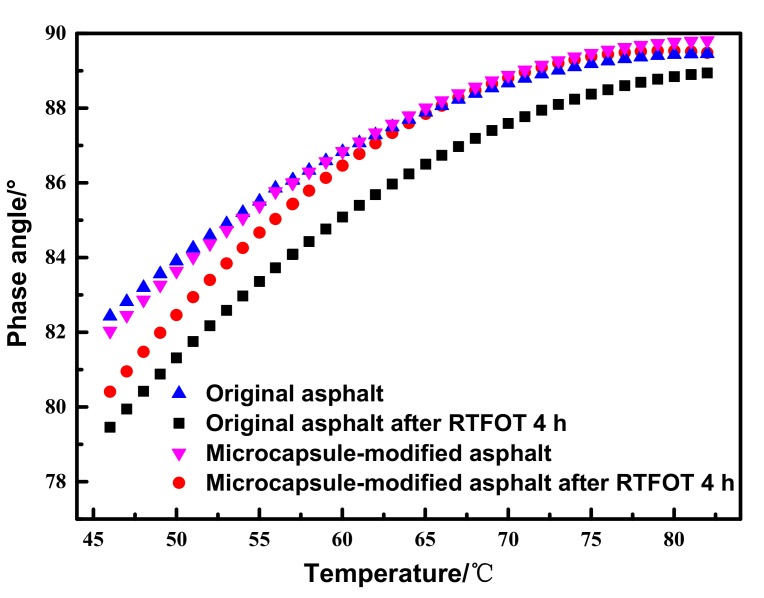
The phase angle of different asphalt samples before and after RTFOT aging.

The results of the phase angle δ before and after RTFOT aging of different asphalt samples are shown in Figure 12. It can be seen that the phase angle δ of asphalt remained almost unchanged after adding microcapsules to original asphalt, indicating that the addition of microcapsules does not change the viscoelasticity of asphalt. With the increase of temperature, the phase angles δ of the four kinds of asphalt increased, which implies that asphalt changed from an elastic state to a viscous state. Compared with the original asphalt samples, the phase angle δ of the aged asphalt samples decreased. The more serious the asphalt aging is, the more the phase angle δ decreases. As shown in Figure 12, the phase angle δ of the microcapsule-modified asphalt after RTFOT 4 h was higher than that of the original asphalt after RTFOT 4 h. The results show that aging of the microcapsule-modified asphalt was less than that of the original asphalt; that is, microcapsules can play the role of slowing down the aging process, which is consistent with the results of the complex shear modulus of asphalt.

## 4. Conclusions

In this work, novel sustained-release microcapsules containing rejuvenator were prepared by the solvent evaporation method. The short-term aging properties of sustained-release microcapsules containing rejuvenator-modified asphalt were studied. The following conclusions were drawn:

(1) The sustained-release microcapsules with a high coating rate can be obtained when sodium dodecyl sulfate (SDS) is used as the emulsifier, and the core/shell mass ratio is 1:1.3. The microcapsules are in complete encapsulation with an average particle size of 60 μm and a smooth surface observed by SEM.
(2) The prepared microcapsules have good thermal stability in the range below 130 °C. Upon further increasing the temperature to 140 °C, the shell surface morphology of the microcapsules becomes coarser, indicating a large number of micropore structures in the shell of the microcapsules. The microporous structure provides favorable conditions for the release of the rejuvenator in asphalt.
(3) In contrast to the 44% mass loss rate of rejuvenator at 185 °C for 4 h, the mass loss rate of rejuvenator coated in microcapsules is only 26% under the same conditions. The release rate of rejuvenator is obviously slowed down by the microcapsules, exhibiting obvious slow release characteristics.
(4) The penetration ratio of microcapsule-modified asphalt after aging is higher than that of original asphalt after aging; the softening point increment of the microcapsule-modified asphalt after aging is lower than that of the original asphalt; the ductility ratio of the microcapsule-modified asphalt is far higher than that of the original asphalt. These results indicate that the addition of sustained-release microcapsules could obviously improve the anti-aging properties of asphalt.
(5) Viscosity aging index and DSR test results show that asphalt modified by sustained-release microcapsules containing rejuvenator possesses excellent anti-aging properties. It is very useful to apply these novel sustained-release microcapsules to modify asphalt and improve its anti-aging ability.

## Figures and Tables

**Table 1:** Properties of original asphalt and modified asphalt.

Properties	Results		Specification
	Original Asphalt	Modified Asphalt	
Penetration (25 °C, 100 g, 5 s) (0.1 mm)	69	68	60–80 (ASTM D5) [19]
Ductility (15 °C, 5 cm/min) (cm)	120	110	≥100 (ASTM D113) [20]
Softening point (°C)	47	47	≥40 (ASTM D36) [21]
Density (25 °C) (g/cm^3^)	1.038	–	–
